# Retention of microplastics by biofilms and their ingestion by protists in rivers

**DOI:** 10.1111/1758-2229.70016

**Published:** 2024-10-09

**Authors:** Leandra Hamann, Jennifer Werner, Felicia J. Haase, Massimo Thiel, Anja Scherwaß, Christian Laforsch, Martin G. J. Löder, Alexander Blanke, Hartmut Arndt

**Affiliations:** ^1^ Bonn Institute for Organismic Biology, Section 2, Animal Diversity University of Bonn Bonn Germany; ^2^ Institute of Zoology University of Cologne Cologne Germany; ^3^ Coastal and Marine Research Centre Griffith University Southport Queensland Australia; ^4^ School of Environment and Science Griffith University Southport Queensland Australia; ^5^ Department Animal Ecology I and BayCEER University of Bayreuth Bayreuth Germany

## Abstract

Microplastics (MPs) are released into the environment through human activities and are transported by rivers from land to sea. Biofilms, which are ubiquitous in aquatic ecosystems such as rivers, may play an essential role in the fate of MPs and their ingestion by biofilm protists. To assess this, biofilms were naturally grown on clay tiles in the River Rhine, Germany, and analysed in a combined field and laboratory study. Compared to the ambient river water, biofilms grown for 6, 12, and 18 months in the River Rhine contained up to 10 times more MPs. Between 70% and 78% of all MPs were smaller than 50 μm. In laboratory experiments, clay tiles covered with 1‐month‐old naturally grown biofilm retained 6–12 times more MPs than clay tiles without biofilm coverage. Furthermore, the ingestion of MPs of 6 and 10 μm by the ciliate Stentor coeruleus was confirmed, and a positive correlation between ingestion rates and ambient MP concentrations was found. The results are relevant for particle transport models in riverine systems, risk assessment of MPs regarding their distribution and fate in the aquatic environment, and the effects of MPs on micro‐ and macroorganisms.

## Graphical abstract

Biofilms, which are ubiquitous in aquatic ecosystems such as rivers, may play an essential role in the fate of microplastics (MPs) and their ingestion by biofilm protists. A combination of field and laboratory experiments shows that riverine biofilms contain up to 10 times more MPs than ambient water and retain 6–12 times more MPs than surfaces without biofilms. The ciliate Stentor coeruleus ingests MPs, especially in high ambient concentrations.

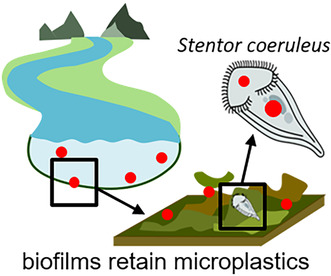

## INTRODUCTION

Small plastic particles, so‐called microplastics (MPs), are released into the environment due to a broad range of human activities, such as the use of cosmetics, abrasion from synthetic clothing, loss of virgin plastic pellets, tyre wear, or fragmentation from larger plastic objects (Auta et al., 2017; Boucher & Friot, 2017; De Falco et al., 2019). As a result, all environmental compartments contain MPs, from the atmosphere (Evangeliou et al., 2020) to soil (Horton et al., 2017), and freshwater and marine environments (Ivar Do Sul & Costa, 2014; Wagner et al., 2014). In marine systems, studies have investigated the connection of MP presence and concentration to its retention and negative ecological effects (Ivar Do Sul & Costa, 2014; Pirsaheb et al., 2020). In freshwater systems, it was shown that rivers play a significant role in transporting and distributing MPs and plastic litter from inland to shores and oceans as their final sink (Lebreton et al., 2017). In the River Yangtze (Asia), on average 4137 MPs per m^3^ were found with 90% of particles in a range of 0.5–5 mm (Zhao et al., 2014), the River Nile (Africa) contained up to 1718 MPs per m^3^ in surface waters (Shabaka et al., 2022). Rivers show MP concentrations that exceed concentrations found in estuaries and marine environments (Luo et al., 2019), especially in urban areas and wastewater effluents (Leslie et al., 2017). However, relatively little is known about their fate and effects in freshwater systems compared to marine ecosystems (Horton et al., 2017; Wagner et al., 2014), which are necessary to quantify potential risks and inform mitigation strategies, such as life cycle assessments (LCA) and political measures (Maga et al., 2022).

In order to close the gap between MPs presence, exposure, ingestion, and possible negative ecological effects riverine systems, we measured MP concentration and investigated MP retention by biofilms and ingestion by protists in the river Rhine. Biofilms grow on every aquatic surface (Sentenac et al., 2022). They consist of microbial communities of bacteria, algae, and protists living in a matrix of extracellular polymeric substances (EPS). Biofilm formation is initiated by bacteria that settle on substrates and excrete EPS, which in turn supports the attachment of algae, protozoans, and metazoans with maturing (Costerton et al., 1981; Vasudevan, 2014). They grow on organic and inorganic substrates (Branda et al., 2005; Böhme, Risse‐Buhl & Küsel, 2009), such as stones, leaf litter, macrophytes, animals, artificial substrates (Arndt et al., 2003) and MPs. With ongoing growth, seasonality, and the entrapping of different species, biofilms form a three‐dimensional structure with varying thicknesses (Vasudevan, 2014). Within river ecosystems, biofilms influence the food chain (Vasudevan, 2014), nutrient cycling (Chen et al., 2020), and degradation of herbicides (Bighiu & Goedkoop, 2021). Therefore, they pose an important study object regarding MPs, retention and effects on single organisms, microbial communities, and trophic transfer in rivers.

In the River Rhine (Europe), 0.5 to >20 MPs per m^3^ were measured with sizes ranging from 300 μm to 5 mm at 11 locations (Mani et al., 2016). Sediments at the shore of the River Rhine contained 228–3763 MPs per kg sediment with MP sizes ranging from 63 μm to 5 mm (Klein et al., 2015). In the River Rhine's floodplains, concentrations of 25,502 up to 84,824 MP per kg dry soil were found in the size range of 11 μm to 5 mm (Rolf et al., 2022). One entry path for MPs is over 3100 wastewater treatment plants along the Rhine that release around 33.9 × 10^12^ MPs per year (Schmidt et al., 2020). This shows that MPs are already present and potentially exposed to ingestion by organisms in the river Rhine. Models of riverine transport of MPs show that the growth of biofilms on MPs influences the settling behaviour of MPs and thus, their distribution in the river water column (Besseling et al., 2017; Hoellein et al., 2019), similar to marine environments (Lobelle & Cunliffe, 2011; Yokota et al., 2017; Zettler et al., 2013). In reverse, the retention of MPs within biofilms is largely unknown and so far not considered as additional environmental stressor or implemented in current MPs retention models (Sentenac et al., 2022; Wang et al., 2023). Biofilms might act as a temporary sink for MPs (Wang et al., 2023) and thereby, foster the incorporation of MPs into microbial and macrobial food webs as it has been observed for other kinds of suspended particles in streams (Arnon et al., 2009; Battin et al., 2016; Böhme et al., 2009). In addition, many organisms have evolved feeding techniques that allow them to discriminate against inert particles such as sand grains and other indigestible particles including plastic particles. However, when plastic particles are inside potential food organisms or embedded inside the biofilm matrix, these selection mechanisms may not work. For example, *Amoeba proteus* ingested larger MPs via prey, which would not have been taken up otherwise (Mondellini et al., 2024). Additionally, protists could surround MPs by a membrane as a mask hiding their indigestible nature (Bulannga & Schmidt, 2024). MP particles could thus potentially enter higher trophic levels via this ‘hidden door’ (Ramsperger et al., 2020).

In order to investigate the effects of MPs presence in riverine ecosystems, its retention by biofilms was investigated in field and laboratory experiments. Thereby, the focus was on particles <100 μm as this particle size is particularly relevant for the inhabiting protists.

Additionally, the ingestion rate of artificial MPs was tested for a model ciliate which is typical for Rhine river biofilm communities, *Stentor coeruleus*, in exposure experiments to determine the role of protists within biofilms and the possible transfer of MPs to biofilm grazers. *Stentor* can contribute up to 75% of ciliate biomass in the biofilm of the River Rhine at Cologne (Ackermann et al., 2011).

## EXPERIMENTAL PROCEDURES

### Study site: River Rhine in Cologne (Germany)

The River Rhine flows from its origin in Switzerland northwards through the west side of Germany and ends in the Dutch North Sea. The stretch of the River Rhine in Cologne, Germany, is characterized as the Lower Rhine (Preusser, 2008) with a flow velocity of 1.5 m s^−1^. The study was conducted at the Ecological Rhine Station of the University of Cologne, a boat permanently anchored in Cologne‐Bayenthal (Rhine‐km 684.5). Behind the boat, a float with channels is attached in the downstream direction, allowing biofilms to grow on different substrates under natural conditions. These channels have a depth of 0.5 m and the water current of the River Rhine is slowed down to an average of 0.8 m s^−1^, representing the conditions of the neighbouring groyne fields (Ackermann et al., 2011).

### Field experiment: Natural MP concentration in biofilms

#### Sample exposition in the river Rhine

In total, 96 clay tiles (4.9 × 4.9 × 0.5 cm) were placed in the float channels of the Ecological Rhine Station in the River Rhine in February 2020 and sampled after three time periods (6, 12, and 18 months). After 6 months of growth and exposure time (September 2020), tiles with biofilms were randomly selected and carefully transferred from 12 of the 96 tiles, using a wooden brush (natural fibres) and 40 mL of unfiltered River Rhine water measured with a glass beaker. Samples were stored in glass bottles covered with aluminium foil in a −18°C freezer until further analysis. The operators were careful not to contaminate the samples with MPs from clothes or atmospheric plastic particles while taking the samples. Brush and glass beaker were rinsed with Rhine water in between each sample taking. In addition, three control samples with 40 mL of River Rhine water were taken in the same manner, including stirring with the brush but without the biofilms, and stored under the same conditions. The same procedure was repeated after 12 months (February 2021) and 18 months (September 2021).

#### Sample analysis

The samples were analysed at a laboratory of the University of Bayreuth. To prevent contamination, all samples were handled in laminar flow boxes (Laminar Flow Box FBS, Spetec GmbH) for analysis. Furthermore, the laboratory was equipped with an air purifier (DustBox with HEPA H14 filter, Möcklinghoff Lufttechnik GmbH). All tools and devices were cleaned twice before and after each usage by rinsing with filtered deionized water and filtered 35% ethanol (Ø 2 and 0.2 μm, respectively). All chemicals, solutions, and liquids were filtered before use. Cotton lab coats were worn during the sample preparation and analysis. Before analysis with Micro‐Fourier transform infrared spectroscopy (μFTIR), the samples were purified with an enzymatic‐oxidative purification protocol based on the method by Löder et al. (2017). Organic matter was destroyed with sequential incubation in sodium dodecyl sulphate and protease, followed by density separation in zinc chloride solution (density 1.7–1.8 g cm^−3^) to remove mineral particles. Depending on the amount of material left, samples were subdivided (a minimum ¼ was used) as described by Rolf et al. (2022) and finally filtered onto Anodisc filters (mesh size 0.2 μm, 25 mm diameter Anodisc, Whatman GE Healthcare) for μFTIR imaging. The samples were measured with a LUMOS II μFTIR spectrometer (Bruker Optics GmbH & Co. KG, Ettlingen, Germany) equipped with a 32 × 32 detector pixel focal plane array (FPA) detector.

Whole sample filters were measured in transmission mode in a wave number range from 3600 to 1250 cm^−1^ with a resolution of 8 cm^−1^ and an accumulation of 2 scans. The background was measured with 36 scans on the pure Anodisc filter. The FPA detector combined with the IR objective results in a spatial resolution of 5.9 μm per pixel. The data was converted to the ENVI file format in the Bruker OPUS Software (version 7.5) imported in the Epina ImageLab software (version 3.47) and the polymer type of each particle identified by a random forest decision classifier software tool for MPs (‘BayreuthParticleFinder’). This classifier can identify 22 different polymers automatically and enables rapid analysis within 0.5 h per filter (Hufnagl et al., 2019; Hufnagl et al., 2022). Each automatically identified MP particle was manually double‐checked against reference spectra according to a four‐eye principle by experienced staff for quality assurance. Subsequently, the particle sizes were measured and shapes were recorded from the photographs of the Anodisc filters. The largest dimension was considered as the particle size.

To compare the MP size distribution to the study of Rolf et al. (2022), their size classes were adapted and complemented by an additional size class at the lowest size spectrum because these small MPs are highly relevant for protists. Thus, the data was analysed with nine size classes: <11, 11–50, 51–100, 101–150, 151–300, 301–500, 501–1000, 1001–5000 and >5000 μm. The particles were also assigned to three shape categories: fragments, fibres, and pixels. Pixels describe particles with only one pixel in diameter and thus, an undetermined shape but with an IR spectrum of MPs.

### Laboratory experiments: Biofilms and protists

#### MP particles for laboratory experiments

For all laboratory experiments, Fluoresbrite® polystyrene (PS) monodispersed microspheres (Polysciences Europe GmbH, Hirschberg an der Bergstrasse, Germany) internally dyed with phycoerythrin (‘polychromatic red’, 525–565 nm excitation, Ø 1 μm, and Ø 6 μm) and fluorescein (‘yellow‐green’, 441–485 nm excitation, Ø 10 μm) were used as MP model particles. As the MPs came in suspension, the MP concentration was diluted to the desired concentration of 500 particles mL^−1^ of 6 μm‐sized particles in the first experiment and 1, 6, and 10 μm‐sized particles in the second experiment, respectively. The MP concentration for the experiments was chosen to be similar to previous exposure experiments (GESAMP, 2016). Due to the fluorescent nature of the used MPs in this experimental study, the operators did not exclusively use plastic‐free materials as this did not impact the quantitative analysis of fluorescent MPs.

#### Experimental setup in endless flow channels

**FIGURE 1:**
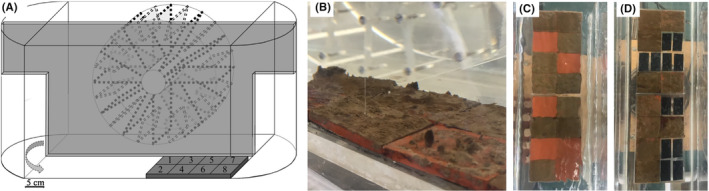
Experimental setup for the biofilm studies in the laboratory. (A) Endless flow channel with a paddle wheel to induce a current and a frame for tiles in the opposite side (picture taken and modified from Schössow et al., 2016). (B) Natural grown biofilms on clay tiles in the endless flow channel. (C) + (D) Clay tiles with biofilms, either in combination with empty clay tiles (C) or empty acrylic tiles (D, experiment 1).

For the simulative flow channel experiments, biofilms were grown prior under natural conditions on clay tiles (4.9 × 4.9 × 0.5 cm) in the flow channels of the Ecological Rhine Station in the River Rhine for 30 days in December 2018 and in March 2019. The aim of the experiment was to compare the retention of MPs by clay tiles covered with biofilms to the retention by surfaces without biofilms. The experimental setup consisted of four endless oval‐shaped flow channels (Figure 1, Schössow et al., 2016), which were equipped with paddle wheels connected to an electromotor to manually adjust the flow velocity. The channels were filled with 10 L of filtered River Rhine (Whatman® GF/C filters, Ø 1.2 μm) and 10 L of regular tap water. Frames were installed to hold the tiles in place on the opposite side to the paddle wheels (Figure 1). The clay tiles with biofilms were inserted 24 h before the experiments in two of the four channels to ensure that the biofilms could acclimate to the water temperature, light conditions, and flow velocities in the setup.

In the first experiment, four substrate types were exposed in the experimental flow channels in addition to the biofilm samples on the rough clay tiles: Rough and smooth acrylic glass tiles, and rough and smooth clay tiles (4.9 × 4.9 × 0.5 cm, Figure 1). In a second experiment, only rough clay tiles with and without biofilms were compared (for experimental details and the number of replicates, see Supplementary Information (SI) Tables SI‐1 and SI‐2). In both experiments, the substrates were exposed for 24 h to the fluorescent MPs under a continuous, simulated stream flow of 0.1 and 0.2 m s^−1^. At the end of experiments, the retained particles on the tiles' surfaces and on biofilms were transferred into 50 mL screw‐cap tubes (Sarstedt, Germany) using a brush and rinsing the tiles with filtered River Rhine water (10 mL for tiles without biofilms, and 25 mL for tiles with biofilms to allow repeated rinsing of tiles and brush). The samples were positioned upright and the volume of sedimented organic matter in the biofilm samples was noted to determine the dilution. All samples were stored in the dark at 4°C until further analysis.

In preparatory studies, confocal laser scanning microscopy was used to see the particle depth within the biofilms. Therefore, biofilms were grown on microscopic slides for 3 months and exposed them to 6 and 10 μm fluorescent MPs for 24 h in the flow channels. Afterwards, we used a Zeiss LSM 510 META microscope (Zeiss, Germany) with 20× oil immersion objectives to identify MPs depth in the biofilms. Both MPs sizes were found to be embedded on average in 350 μm depth. There were no significant differences between the samples regarding particle size or flow velocity (total *N* of 6 μm MPs = 280, total *N* of 10 μm MPs = 274). Subsequently, the biofilm samples were destroyed and MPs were quantified visually in the experiments for this study.MP concentrations of samples were quantitatively analysed using an Axiostar Plus Fluorescence (FL) microscope (Carl Zeiss Jena GmbH, 07740 Jena, Germany), which was connected to an external lightning unit (HXP‐120, Zeiss, Germany) for FL excitation and excitation light of the filter set 38 HE as recommended by the MPs manufacturer (excitation BP470/40, beam Splitter FT 495, emission BP 525/50, Zeiss, Germany). Concentrations of MPs in biofilms were determined by quantitatively counting the fluorescent MPs particles in 2 μL aliquots of the well‐mixed sample placed on microscopic slides, in triplicates per sample. MP counts per cm^2^ of tiles with biofilms were calculated by taking the mean value of the triplicates multiplied by the dilution factor (biofilm volume of total sample volume) and dividing by the tiles' surface area. MP counts per cm^2^ of tiles without biofilms was assumed to have a dilution factor of 0.02401 mL (area of tiles multiplied by maximum height of MP particles, i.e., 10 μm).

The hydrodynamic conditions were calculated based on Arnon et al. (2009). The mean flow rate was calculated with Q=U*w*d for each flow velocity *U* of 0.1 and 0.2 m s^−1^, the tank width *w* of 0.14 m, and water column depth *d* of 0.1 m. Froude number was calculated with *Fr* = *U*/$\sqrt{g*d}$, with *g* as the gravitational constant 9.81 m s^−2^ and the water column depth *d*. When the Froude number indicates slow flow (*Fr* <1), tank represents natural flow conditions in rivers (Boavida et al., 2011; Kuriqi & Ardiçliolu, 2018). The Reynolds number was calculated for the three particle sizes and two flow velocities to be between 0.0996 and 1.992, thus, Stokes' law was applied to calculate the settling velocities of the particles:

$$v=\frac{2\;\left(\mathrm{\rho p}-\mathrm{\rho w}\right)}{9*\mu }*g*r2,$$

with *ρ*_*p*_ as the density of the particles (PS, 1050 kg m^−3^), *ρ*_*w*_ of the fluid (999.7 kg/m^3^), *μ* as the dynamic viscosity of water (1.307 × 10^−3^ kg m^−1^ s at 10°C, measured for the first experiment) and *r* as the particle radius (Table 1).

**TABLE 1:** Average hydrodynamic conditions in the experiments.

Flow tank velocity	*U* (m s^−1^)	0.1	0.2
Flow tank width	*w* (m)	0.14	0.14
Water column depth	*d* (m)	0.1	0.1
Mean volume flow rate	*Q* (m^3^ s^−1^)	0.0014	0.0028
Froude number	*Fr*	0.10096	0.20193

Particle settling velocity		m s^−1^	m 24 h^−1^
For 1 μm	*v*	1.894 × 10^−7^	0.0164
For 6 μm	*v*	3.397 × 10^−6^	0.293
For 10 μm	*v*	1.047 × 10^−5^	0.904

*Note*: Mean flow rate and Froude number are calculated for the two flow velocities tested (0.1 and 0.2 m s^−1^). The particle settling velocities are calculated for all particle sizes and given in m s^−1^ and for the total duration (24 h) of the experiment.

To determine sedimentation and recovery rate of the particles after 3 h and at the end of the experiments, water samples were taken in triplicates per sample spot in front, above, and behind the frames during the second set of experiments in all four tanks. A syringe with a broad opening was used and samples were filled into 15 mL graduated centrifuge tubes (Sarstedt, Germany). For each sample, triplicate aliquots of 5 μm each were counted in the same manner as the biofilm samples. The mean concentration of all samples was extrapolated to the water volume for each tank. The recovery rate was estimated using the sum of all particles found in biofilm and water samples by accounting for their total volumes.

#### MP uptake by protists

The uptake of MPs was studied for the biofilm‐associated ciliate *Stentor coeruleus* (Stentoridae), which is an omnivorous filter‐feeder (Fenchel, 1987). *Stentor* was chosen for the experiments because of its occurrence in the River Rhine in Cologne and its large size of 250 μm–1.5 mm (Ackermann et al., 2011), which facilitates optical analysis of MP uptake. Cultures of *S. coeruleus* were obtained from Helbig (Prien am Chiemsee, Germany) and stored at 20°C under laboratory conditions. The cultures were fed with *Chlorococcum* sp. (Chlorococcaceae; ‘Lebendkulturen Helbig’, Prien am Chiemsee, Germany) and *Chlamydomonas asymmetrica* (Chlamydomonadaceae; Culture collection of algae of the University of Cologne (CCAC)) on every third to fourth day.

Non‐axenic Stentor cultures were pre‐grown for 1 week in autoclaved River Rhine water supplied with wheat grain. The ingestion of MPs by *S. coeruleus* was studied for 6 and 10 μm particles with varying MPs concentrations (500, 1250, 2500, 5000 p mL^−1^, controls: 0 p mL^−1^). The MPs suspension was vortexed prior to the experiment to avoid aggregation. Before each experiment, 70 mL of autoclaved Rhine river water with MPs and 15 mL of the *S. coeruleus* cultures were transferred into 120 mL tubes. Bacteria (about 10^6^ cells mL^−1^) were present in the experiments to mimic natural conditions. Stentor behaved thigmotactic as in the natural environment in the Rhine biofilms. The idea of the experiments was to test, whether the MPs are principally taken up and which concentrations could potentially be reached within individuals of a ciliate species which is abundant on River Rhine biofilms (up to 2000 stentorids cm^−2^ of biofilm; Ackermann et al., 2011). In the experiments, between 10 and 20 ciliates mL^−1^ were present. Experiments were run in triplicates for 1 h for each concentration. To prevent sedimentation of particles, experimental tubes were attached to a plankton wheel (2.39 rpm). After the experiments, 1 mL was taken from the 120 mL tubes and immediately transferred to a Sedgewick‐Rafter cell. All *S. coeruleus* specimens were immediately examined alive and individually for MPs uptake under the microscope (10× magnification with fluorescent light, Axiophot Fluorescent Microscope, ZEISS). The immediate assessment of the MPs uptake is important because fixation methods might lead to egestion and underestimation of the ingested MP amount (Pace & Bailiff, 1987).

### Statistical analyses

The data of the field and laboratory experiment were analysed in the R programming environment (version 3.5.2, R Core Group 2018, packages: ggplot; Wickham, 2016), dunn.test (Dinno, 2024) to identify significant differences between MP concentrations in biofilms and the ambient River Rhine water as controls, the influence of different substrates on MPs concentration, and influence of ambient MP concentration on the ingestion of MPs by ciliates. Before data analysis, the data structure was checked for normality, heteroscedasticity, and outliers (Zuur et al., 2010). Mean concentrations between samples were tested for significance using *t*‐test or ANOVA as a parametric test or alternatively a Kruskal–Wallis‐test as a non‐parametric test. Data are reported as the mean value with standard deviation. The control groups, in which no MP particles were added, were zero in all experiments and thus, were not included in the statistical tests.

## RESULTS

### Field experiments: Natural MP concentrations in biofilms

**FIGURE 2:**
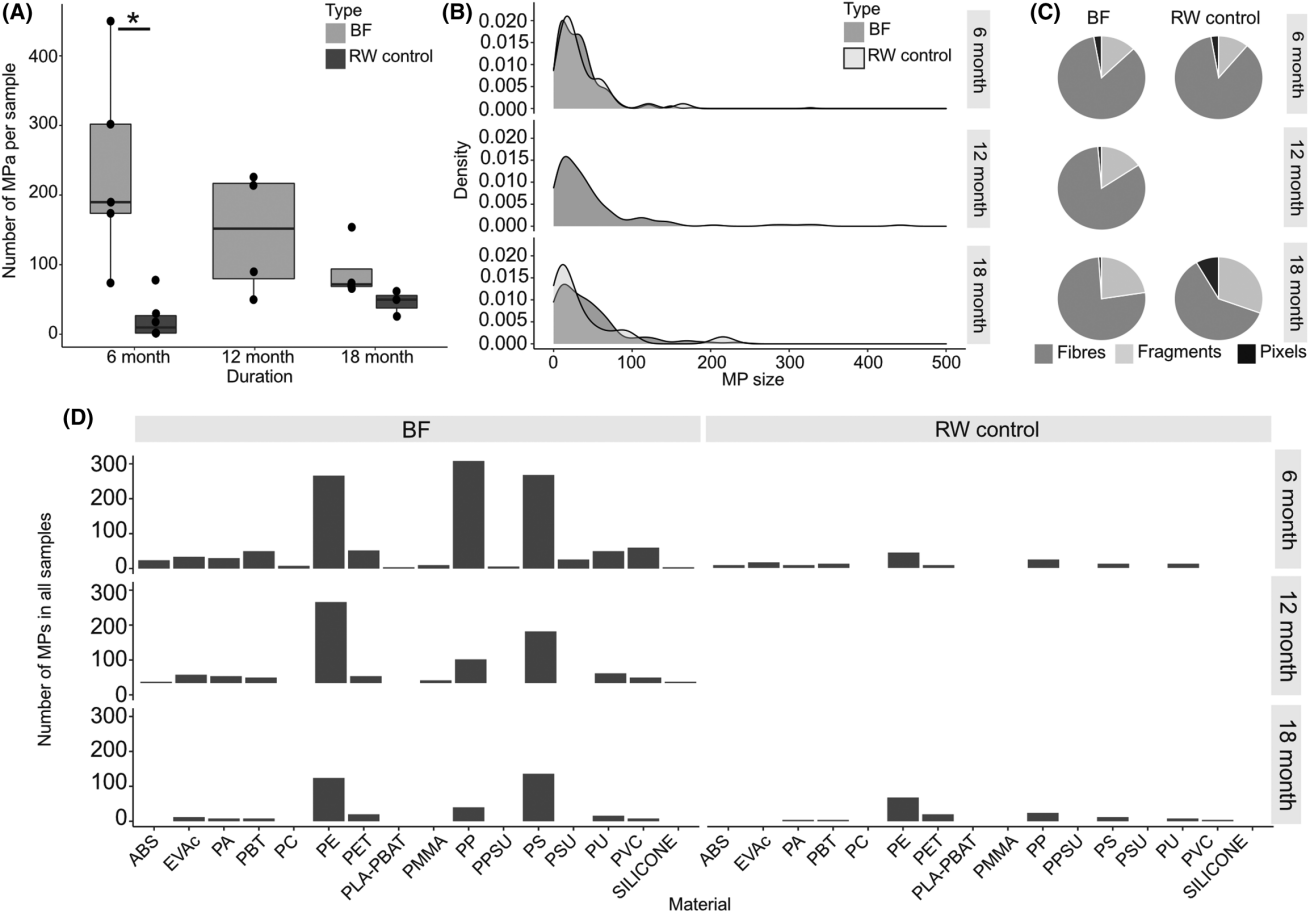
MPs found in the field samples of biofilms (BF) and river water (RW) controls across the duration of the experiment after 6, 12, and 18 months of growth in the River Rhine. (A). Number of MP particles found in each sample (black points) for biofilms and the RW controls, which are samples taken from the ambient water. Significances (* = *p* <0.05) were identified with Kruskall–Wallis and post‐hoc Dunn test (method ‘holm’). (B) Density plot of the particle sizes found in biofilms and RW controls for each duration for particles <500 μm. (C) Relative share of particle shapes distinguished for fragments, fibres and pixels found (pixel indicate particles of a size that is one pixel, thus, no shape could be determined, see text). (D) Total number of MPs particles across all samples for each plastic material across all samples of biofilms and RW controls, respectively.

After 6 months in the River Rhine, the biofilm samples diluted in 40 mL of filtered Rhine water contain a mean value of 240 ± 143 MPs (*n* = 5, Figure 2A). This value is significantly higher than the amount of MPs in the control samples with the same amount of river water (40 mL) but without biofilms (24 ± 30 MPs, *n* = 4, *p* < 0.05, Kruskall–Wallis and post‐hoc Dunn test (method ‘holm’)). In the 12‐month‐old biofilm samples, the average concentration is 147 ± 88 MPs (*n* = 4) and in the 18‐month‐old biofilm samples, the concentration is 93 MPs (*n* = 4). This is not significantly different from the corresponding controls with 48 ± 18 MPs per 40 mL Rhine water (Kruskall–Wallis and post‐hoc Dunn test (method ‘holm’), *n* = 3). Note here that during the transportation of the frozen samples by a transportation company to the University of Bayreuth for analysis, several samples were destroyed, which led to the varying number of replicates and the total loss of the control samples after 12 months. By converting MP concentration in the biofilm samples to the tiles' surface area, the average MP abundance ranges between 4 and 10 MPs cm^−2^ of biofilm‐covered surface in the size range of 5.9–5000 μm. The thickness of biofilms in river water varies between 140 and 160 μm after 40 days (Roche et al., 2017) to a maximum of several mm for longer growth periods (Arnon et al., 2009; Zhang & Bishop, 1994). In the absence of direct measurements of the biofilm thickness during our study, we assumed an average biofilm thickness of 1 mm with a density close to water, the calculated concentration per weight ranges between 38,734 and 99,958 MP kg^−1^, depending on experimental duration.

MP size distribution is similar in all biofilms and river water samples after 6, 12, and 18 months (Figure 2B). On average, 73% and 76% of MPs are smaller than 50 μm, and between 20% and 24% are smaller than 11 μm in biofilms and river water samples, respectively (Supplementary Table SI‐2). An average 3.6% of the particles are larger than 300 μm in the biofilm samples. The river water samples contain no particles larger than 300 μm. MP properties regarding shape and material are similar in biofilms compared to the Rhine water controls (Figure 2C). Fragments accounted for the majority of MP shape, ranging between 76.3% and 84.3% in the biofilm samples, while pixels range between 12.8% and 22.6%, and fibres between 1.1% and 2.8%. In the river water samples, 61.1% and 86.1% are fragments, 11.1%, and 30.5% are pixels, and 2.8% and 8.3% are fibres. In total, 16 different polymer types were identified across all samples (Figure 2D). The most frequent polymers are polyethylene (PE), polypropylene (PP), and PS.

### *Laboratory experiment: Retention of MPs by biofilms and the ciliate* Stentor coeruleus

**FIGURE 3:**
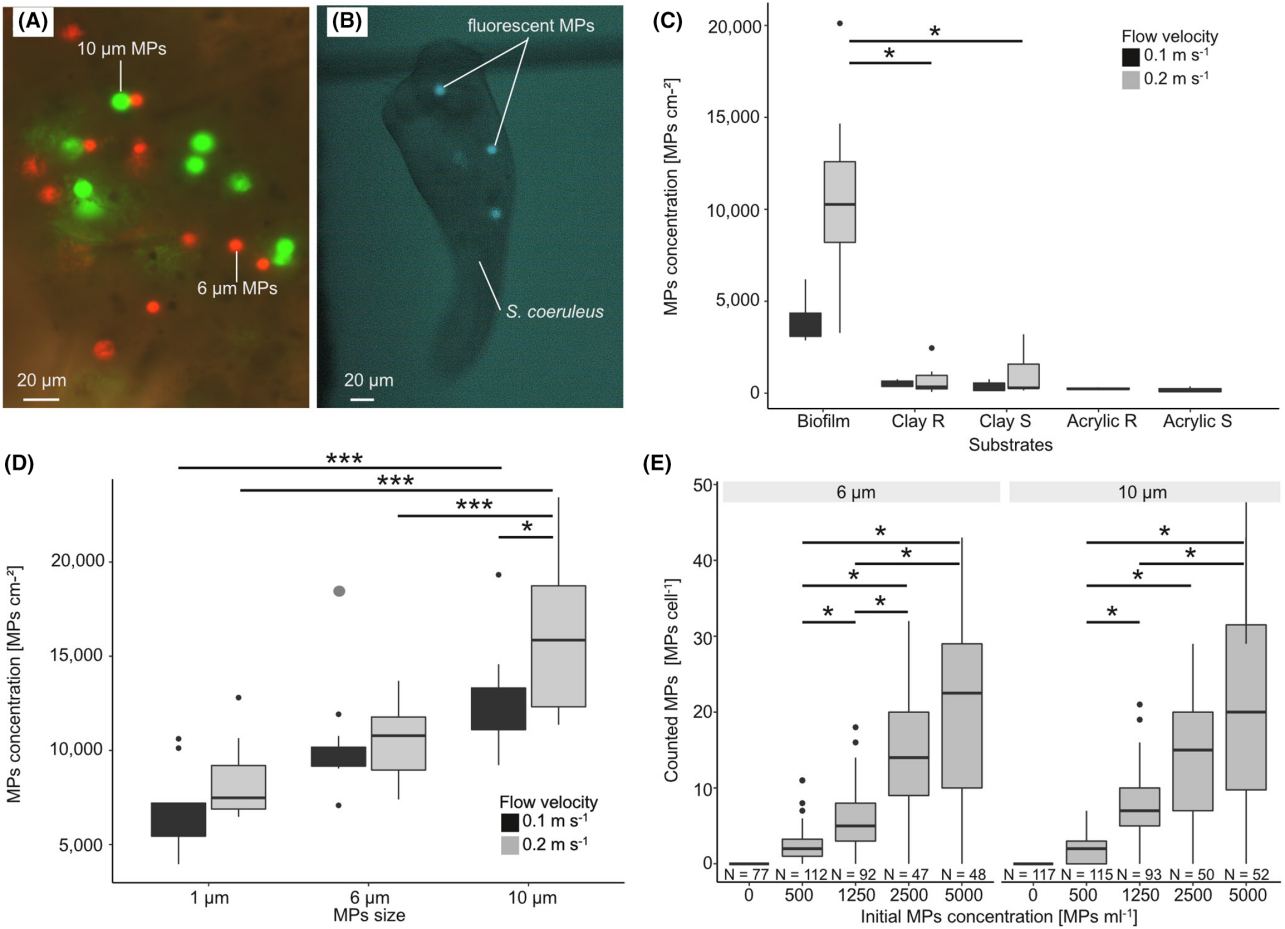
Laboratory experiments in flow tanks with biofilms grown in the River Rhine and fluorescent MPs. (A) Fluorescent microplastics of 6 μm (red) and 10 μm (green) in biofilm samples (Fluorescent microscope picture, ×20 magnification). (B) *S. coeruleus* with 10 μm green plastic particles (epifluorescent microscope). (C) Comparison of the particle retention of the 6 μm particle size by the substrates biofilm, rough clay tiles (R), smooth clay tiles (S), rough acrylic (R), and smooth acrylic (S) for two different flow velocities (0.1 m s^−1^ (black) and 0.2 m s^−1^ (grey)) after exposition for 24 h. Kruskal–Wallis‐test (* = *p* <0.05), post‐hoc Dunn‐test with method ‘holm’. (D) Concentration of microplastics (MP cm^−2^) in the biofilms compared for the three MP sizes (1, 6, and 10 μm) for two flow velocities (0.1 m s^−1^ (black) and 0.2 m s^−1^ (grey)) after 24 h. One outlier was removed from statistics (grey circle), ANOVA *p* <0.05, post‐hoc TukeyHSD. * < 0.05, *** < 0.001. (D) Ingestion of MP (MP cell^−1^) for the particle sizes of 6 and 10 μm by *S. coeruleus* in presence of varying concentrations (MP mL^1^). Kruskal–Wallis‐test, post‐hoc Dunn‐test (* = *p* <0.05). The control (concentration of 0 MP mL^−1^) was significantly different from all other groups (not indicated).

In the lab experiments, the fluorescent MP particles were easily countable in the biofilm samples (Figure 3A) and the cell bodies of *S. coeruleus* (Figure 3B). The retention of fluorescent MPs is 6–8 times higher in biofilms than on rough and smooth clay tiles at a velocity of 0.1 m s^−1^ and 9–12 times higher at a velocity of 0.2 m s^−1^. Statistical analysis shows that for both tested flow velocities the MP retention by biofilms versus by clay tile surfaces without biofilm coverage was significantly higher (Kruskal–Wallis‐test (* = *p* < 0.05), post‐hoc Dunn‐test with method ‘holm’, Figure 3C). The acrylic tiles retain the least MPs with 370 MPs m^−2^ for rough and 305 MPs cm^−2^ for smooth acrylic tiles at a flow velocity of 0.1 m s^−1^. No significant statistical differences are found in MPs retention between the two flow velocities (0.1, 0.2 m s^−1^) in any of the tile types with or without MPs (Figure 3C). Particle size significantly influences the number of MPs retained in the biofilms, that is, the number of retained MPs increases with increasing particle size (*p* < 0.001; Figure 3D). MP concentration in the biofilm samples is at least twice as high for the 10 μm particles as for the 1 μm particles, and it differs significantly between the two flow velocities with 12,639 MPs cm^−2^ for 0.1 m s^−1^ and 16,164 MP cm^−2^ for 0.2 m s^−1^ with the 10 μm particles (*p* < 0.05, Figure 3D). The particle settling velocities are calculated for the duration of the experiments as 0.0164 m 24 h^−1^, 0.293 m 24 h^−1^, and 0.904 m 24 h^−1^ for 1, 6, and 10 μm particles, respectively (Table 1). This means that especially the larger particles could have settled to the bottom of the tank within the experimental duration. This is confirmed by water samples that show a reduction of mean particle concentration to around 266 MPs mL^−1^ after 3 h for the 6 μm MPs (*N* = 24). After 24 h, the concentration further significantly drops to 138 MPs mL^−1^ (*N* = 24, *p* >0.05). The mean volume flow rate in the flow tanks was around 0.0014 m^3^ s^−1^ for 0.1 m s^−1^ and 0.0028 m^3^ s^−1^ for 0.2 m s^−1^ during the experiments (Table 1) and the Froude number indicates slow flow (*Fr* <1). On average, across all particle sizes, particle recovery was 42.64% ± 15.4% after the experiment, slightly less than Arnon et al. (2009) presented in their flume experiments with 50%–70% particle recovery. Based on the low MP retention of acrylic tiles, it is assumed that the surface of the flow channel does not retain a high amount of MPs.

After the exposure to MPs for 1 h, 88.2% of *S. coeruleus* cells showed ingested MP particles. Almost every increase in the exposure concentration leads to a significant increase in particle concentration in *S. coeruleus* for 6 and 10 μm MPs (Figure 3E). The highest amount of ingested MPs is at an exposure concentration of 5000 MPs mL^−1^ with 20.6 ± 12.2 MPs Ind^−1^ for the 6 μm and 21.4 ± 13.4 MPs Ind^−1^ for the 10 μm particles. There are no significant differences related to particle sizes, suggesting that there was no preference for ingestion of the two tested MP sizes (6 and 10 μm).

## DISCUSSION

The field and experimental studies show that biofilms in riverine systems may retain and accumulate MPs, which potentially increases the bioavailability for the incorporated and grazing microorganisms. In the laboratory study, the biofilm‐covered surfaces retain 6 to 12 times more MPs than acrylic or ceramic surfaces. In the river Rhine, MP concentration is up to 10 times higher in natural biofilms than in the ambient water controls. Across the three sampling dates, the biofilm samples contained on average 160 MPs per 24 cm^2^ tile, which equals around 6 × 10^5^ MPs m^2^. This is up to two orders of magnitude larger than found in biofilms in the CaoE River in China (Wang et al., 2023). However, we assume that Wang et al. (2023) underestimated MPs concentrations in biofilms since they did not investigate MPs size below 0.5 mm in detail. The results of this study show that river water controls contain 32 ± 27 MPs per 40 mL, which equals around 800,000 MPs m^−3^, which is much higher than found by other authors in the Rhine river. For comparison, Mani et al. (2016) analysed the MP profile along the River Rhine and reported around 2–6 MPs m^−3^ of Rhine water ranging from 300 μm to 5 mm in size in the area of Cologne, Schrank et al. (2022) found 0.7 to 354.9 MPs m^−3^ in size range of 20–5000 μm on different sample spots of surface waters along the river Rhine, while Erni‐Cassola et al. (2024) measured 4.48 MPs m^−3^ in the size range of 50–3000 μm over 22 months in the Rhine near Basel. The water controls are 100–200‐fold higher than the highest MP concentrations found in rivers in Asia (Luo et al., 2019; Strady et al., 2021) and 4 times higher as found in urban canals in Amsterdam and effluents from wastewater treatment plants (Leslie et al., 2017). This can be explained, on one hand, by potential contamination of the samples during handling and analysis. However, assuming that biofilm and water control samples were contaminated equally, the biofilm samples show a much higher MPs concentration. On the other hand, this study analysed MPs down to a size of 5.9 μm, a lower cut‐off point than in the previous studies that used a sampling mesh size of 80 μm (Strady et al., 2021), 20 μm (Luo et al., 2019), and 10 μm (Leslie et al., 2017). Depending on the sampled MPs sizes, the majority of MPs might not be identified, as around 91% of the MPs found in water and biofilm samples were smaller than 100 μm. A similar size distribution was found by Rolf et al. (2022), who reported that 75% of MPs are in the size of 11–150 μm in floodplains in the Cologne area in 2019 and the MPs found in the River Elbe (Germany), in which 96% of MPs in the water column were smaller than 20 μm with the smallest analysed diameter of 4 μm (Triebskorn et al., 2019). Also, MPs shape and material align with the MPs concentration found in the Rhine water (Erni‐Cassola et al., 2024), the Rhine's floodplain soils (Rolf et al., 2022), and the River Elbe in Germany (Schrank et al., 2022; Triebskorn et al., 2019). The comparison across rivers suggests that studies with a higher cut‐off point highly underestimate MP concentration and/or that the river Rhine is particularly contaminated with MPs in the size range of 5.9–100 μm, a particle size range that is relevant for many protists (Bulannga & Schmidt, 2024).

The main mechanism for MP retention by biofilms is the sorption of particles due the chemical properties of the EPS matrix and the mechanical MP particle separation by the biofilm surface morphology (Eisenmann et al., 2001) that shows complex three‐dimensional structures with pits, grooves, and caves that increase the surface area (Böhme et al., 2009; Drury et al., 1993; Mikos & Peppas, 1990; Okabe et al., 1997; Roche et al., 2017). It was previously suggested that larger particles are retained through sedimentation, particle interception, and surface straining (Arnon et al., 2009) and that higher flow velocities push particles deeper into the three‐dimensional structure of the biofilms where they are entrapped (De Beer et al., 1996; Reynolds & Carling, 1991; Risse‐Buhl & Küsel, 2009). This supports the finding that the highest MPs concentration in biofilms was found for the 10 μm MPs under the higher flow velocity. Similar to the MPs in biofilms found in the CaoE river in China (Wang et al., 2023), this study did not find major differences in MPs shape, size distribution, or material in water compared to water. Hence, it can be assumed that the retention mechanism of MPs is similar to the entrapment of natural particles and that there is no selectivity for specific MPs types.

The morphology of biofilms, the different particle retention mechanisms, and the changing environmental conditions are an explanation of the high data variance in the field experiments. This can be seen when comparing the sample dates and also in the data variance at one sample date, for example, in the 6‐month samples (Figure 2A). One reason might be the heterogeneous morphology of biofilms that changes with season, location, and temperature, leading to differences in coverage, thickness, and composition that in return affect particle retention (Figure 1B, and, for example, Walker et al., 2013, Wang et al., 2023). For example, ciliates like peritrichs are likely to occur during colder months, whereas heterotrichs, mainly the omnivorous stentorids are found during warmer periods when algae peak, which is this group's main food source (Ackermann et al., 2011). However, the samples taken after 6 and 18 months show high differences in MPs concentrations even though weather data for the 14 days before sample extraction indicate that temperature and rainfall were similar in September 2020 and September 2021 (Supplementary Figure SI‐1). Therefore, biofilms might have been exposed to temporally high MP concentrations before the sampling date. On the sampling date, MP concentrations in the biofilms were still high, while MP concentration in the river water controls had decreased again. The same could explain the detection of MPs larger than 300 μm inside the biofilms, which were not found in the ambient water controls. Local MP concentrations in rivers can vary up to 8 fold (Stanton et al., 2020) or even up to 30 fold (Moses et al., 2023). In the Rhine near Basel, it was found that MPs concentration varied by two orders of magnitude over 22 months (Erni‐Cassola et al., 2024). The small number of water samples acts as contamination controls for the biofilm samples in this study and can only represent a snapshot of changing MP concentrations. MPs abundance is mostly influenced by population density, the number of drainage systems from wastewater treatment plants leading into the river, and heavy rainfalls that wash off litter and MPs from roads, river discharge, and urban surfaces (Erni‐Cassola et al., 2024; Mani et al., 2016; Siegfried et al., 2017; Zhao et al., 2014).

The difference in MP concentrations in water controls and biofilm samples found in this study supports the hypothesis that biofilms might act as a temporal sink for MPs, as also suggested previously by Wang et al. (2023). In previous laboratory studies, plastic particles were found in the depth of biofilms within 90 min of exposure and remained there for 20 days or longer (Stoodley et al., 1994; Okabe et al., 1997). MPs released by biofilms can occur when mature biofilms reach a thickness that cannot withstand the forces of river flow velocities. Parts of the biofilms can tear off, also called sloughing (Wanner & Gujer, 1986; Okabe et al., 1997), which leads to MPs redistribution (Drury et al., 1993). Repeated retention and release by biofilms might significantly change the fate and residence time of MPs in environmental compartments, especially in a presumably highly dynamic system like rivers.

As a non‐selective suspension feeder that prefers bacteria and algae of 7–22 μm in size (Foissner et al., 1992; Wenzel & Liebsch, 1975), *S. coeruleus* shows an expected MP ingestion in the size of 6 and 10 μm, similar to other protists that were found to ingest MPs. *Oxyrrhis marina*, a heterotrophic dinoflagellate, ingested MPs of 7.3 μm, but no larger particles in laboratory studies (Cole et al., 2013). Between 55 and 85 MPs were found per cell in the pelagic ciliate *Tintinnopsis lobiancoi*, higher than MPs uptake under the same conditions by other zooplankton taxa tested such as copepoda, cladocera, polychaeta, rotifera, or mysida (Setälä et al., 2014). Two holotrich ciliates isolated from South African streams ingested MPs particles at the same rate as microbial prey of similar size with up to 3870 MPs h^−1^ (Bulannga & Schmidt, 2022). Two freshwater Ciliophora (*Tetrahymena pyriformis* and *Paramecium caudatum*) and one Amoebozoa (*Amoeba proteus*) showed species‐specific preferences for MPs size (Mondellini et al., 2024). Before the more recent interest in MPs and protozoans, researchers have used artificial particles already in the 60s, 70s, and 80s to study feeding types, clearance rate, bacterivory, or cyclosis in ciliates, flagellates and amoeba (examples are Mueller et al., 1965; Weisman & Korn, 1967; Batz & Wunderlich, 1976; Fenchel, 1980; Borsheim, 1984; Jonsson, 1986; McManus & Fuhrman, 1986; Sherr et al., 1987). Heterotrophic flagellates (Pace & Bailiff, 1987) and pelagic protists (Batz & Wunderlich, 1976; Jonsson, 1986; McManus & Fuhrman, 1986) show a steady state of particle ingestion and egestion after a while. In a study by Eisenmann et al. (2001), the ciliate *Epistylis* ingested up to 1000 particles per individual after 3.5 h of the 24 h experiment with biofilms. After this peak, the number of ingested particles per ciliate decreased rapidly until they did not contain any particles at the end of the experiment. Instead, particles were attached to the stalks of these ciliates (Eisenmann et al., 2001). Regarding a potential selectivity of MPs, Sherr et al. (1987) compared the uptake of fluorescent‐labelled bacteria and fluorescent latex particles by ciliates and flagellates, which showed that the bacteria‐to‐latex particle uptake ratios were 10:1 for the ciliates and 6:1 for the flagellates. Hence, bacteria were preferred over plastic particles for both groups. These results indicate a selection ability of protists to distinguish between different particle types and suggest a slight discrimination of particles of bad food quality (McManus & Fuhrman, 1986; Sherr et al., 1987; Winiecka‐Krusnell et al., 2009). Fenchel (1980) showed particle discrimination by size in suspension‐feeding protozoans due to the functionality of the mouth apparatus, but no discrimination for particle type. Subsequent studies showed that particle uptake by ciliates correlates with particle concentration and particle size, the ladder also depends on cell size of the protozoans (Jonsson, 1986). In conclusion from the present and previous studies, all non‐selective, suspension‐feeding, and filter‐feeding protists in biofilms are likely to ingest MPs. However, the extent of toxicological or ecological effects through MP ingestion by prostists still remains open. The benthic foraminiferan *Haynesina germanica* was exposed to virgin PP pellets for 10 h, which resulted in no lethal or short‐term effects on locomotion or metabolism (Langlet et al., 2020). Other studies showed that the ingestion of MPs may influence abundance, biomass, swimming behaviour, and oxidative stress in ciliates (Bulannga & Schmidt, 2024). Experiments with the marine ciliate *Uronema marinum* showed that an increase of the ambient MPs concentration leads to an increase of the negative effects (Zhang et al., 2021). This indicates that the MP accumulation ability of biofilms may pose its inhabiting protists at higher risks for negative effects. Since biofilms also play an important role in plants, animals, and humans, for example on leaves, teeth, skin, and gut tissue, MPs accumulation may also effect these interfaces (Sentenac et al., 2022).

In the future, more studies and especially long‐term exposure experiments should be conducted with a diversity of protist species and feeding types. Thereby, MPs surface properties could be of particular interest. Surface properties are essential for predatory ciliates to identify prey and influence the ingestion rate (Dürichen et al., 2016; Kersnowska et al., 1988; Ramsperger et al., 2020; Sanders, 1988). In experiments with nanomaterials, surface charges affected bioavailability (Zhu et al., 2013) and hence might pose a relevant selection factor in addition to abundance, size, shape, aggregation, age, colour, and density as shown for marine zooplankton species (Botterell et al., 2020). Similarly, the adsorption of organic matter onto plastics, the so‐called eco‐corona (Shi et al., 2023), or the settlement of microbial organisms can alter physical and chemical surface properties (Yokota et al., 2017). Another effect might be posed by plastic additives that leach during the degradation processes of litter. MP leachates did not show lethal or adverse effects on the activity in benthic foraminiferans (Langlet et al., 2020) but caused oxidative stress when ingested into cells‐based bioassays used for eco‐toxicological effects (Rummel et al., 2019). As biological systems are dynamic systems, effects on single protists might lead to ecological consequences to the whole biofilm community. For example, it was found that algae growth in biofilms was significantly lower when biofilms grew on polyethene or polyethene terephthalate, which then affected biofilm‐grazing snails (Michler‐Kozma et al., 2022), and biofilm community varied depending on glass or plastic substrate in fresh‐ and wastewater (Parrish & Fahrenfeld, 2019). It can be speculated that this effect might also occur when MPs are incorporated into biofilms.

Additionally, the role of protists in MPs retention from the ambient water and the transfer of MPs retained by the biofilm matrix to protists and further trophic levels need to be analysed and quantified. Understanding and quantifying these mechanisms in environmental compartments is crucial to inform and develop models that include fate and exposure rates to assess potential risks associated with MPs (Bulannga & Schmidt, 2024; Maga et al., 2022).

## CONCLUSION

Biofilms naturally grown in the River Rhine in Cologne for three time periods (6, 12, and 18 months) contained MPs of which 70%–78% were smaller in size than 50 μm. MP concentrations in biofilms of the River Rhine were generally higher than in the ambient river water control samples, which indicates that biofilms might act as traps and temporal sinks for MPs in freshwater watercourses. In simulative experiments biofilm‐covered surfaces retain up to 10 times more MPs than surfaces without biofilms, supporting the important role biofilms may play in the retention of MP in aquatic systems. Environmental factors, such as MP concentration and size, seasonal changes, and flow velocity influenced particle retention in this study. Thebiofilm‐inhabiting ciliate *Stentor coeruleus* ingests MPs of the sizes 6 and 10 μm with no preference for either size but showed increased ingestion with increasing ambient MPs concentration. To date, research has mostly focused on vertebrates and invertebrates, and there is a general lack of information on the exposure and toxicological effects of MPs in biofilm macro‐ and microorganisms such as protists. The lack of data allows no conclusion on ecological or toxicological effects at this stage, but the presence of MPs down to a size of 5.9 μm in the River Rhine and the ingestion of MPs in this size range by a common protist reported in this study suggests that MPs are likely to enter the food web at lower trophic levels than previously suggested. More research is required to test the effects of MPs and various polymer types, shapes, sizes, and surface properties on single protists and microbial communities to estimate ecological risks in aquatic environments. Additionally, a revision of distribution models of MPs from land to sea might be needed to account for biofilm surfaces as potentially important MP sinks and as a vector for MPs to enter the food web.

## AUTHOR CONTRIBUTIONS

**Leandra Hamann:** Conceptualization (equal); data curation (equal); formal analysis (equal); investigation (equal); methodology (equal); software (equal); visualization (lead); writing – original draft (lead); writing – review and editing (equal). **Jennifer Werner:** Conceptualization (equal); formal analysis (equal); investigation (equal); methodology (equal); validation (equal); writing – original draft (equal). **Felicia J. Haase:** Formal analysis (equal); investigation (equal); visualization (equal); writing – review and editing (equal). **Massimo Thiel:** Formal analysis (equal); investigation (equal); writing – review and editing (equal). **Anja Scherwaß:** Conceptualization (equal); formal analysis (equal); methodology (equal); writing – review and editing (equal). **Christian Laforsch:** Resources (equal); supervision (equal); writing – review and editing (equal). **Martin G. J. Löder:** Formal analysis (equal); investigation (equal); writing – review and editing (equal). **Alexander Blanke:** Project administration (equal); resources (equal); supervision (equal); writing – review and editing (equal). **Hartmut Arndt:** Conceptualization (equal); methodology (equal); project administration (equal); resources (equal); supervision (equal); writing – review and editing (equal).

## CONFLICT OF INTEREST STATEMENT

The authors declare no conflicts of interest.

## Supporting information


**Data S1:** Supporting Information

**Data S2:** Supporting Information

## Data Availability

The data that supports the findings of this study are available in the supplementary material of this article.
