# Supplementary material for: Retention of microplastics by biofilms and their ingestion by protists in rivers
Source: Environ Microbiol Rep. 2024 Oct 9;16(5):e70016. doi: 10.1111/1758-2229.70016 (PMC11464032; doi:10.1111/1758-2229.70016)
Supplement: Supplementary file 1 — Data S1: Supporting Information [file EMI4-16-e70016-s001.docx]

Supplementary Information

Retention of Microplastics by Biofilms and Ingestion by Protists in Rivers

Leandra Hamann^1,2,3^, Jennifer Werner^1^, Felicia J. Haase^1,4,5^, Massimo Thiel^1^, Anja Scherwaß^1^, Christian Laforsch^6^, Martin G.J. Löder^6^, Alexander Blanke^2^, Hartmut Arndt^1^

^1^ Institute of Zoology, University of Cologne, Cologne, Germany

^2^ Bonn Institute for Organismic Biology, Section 2, Animal Diversity, University of Bonn, Bonn, Germany

^3^ Whitney Laboratory for Marine Biosciences, University of Florida, Saint Augustine, USA

^4^ Coastal and Marine Research Centre, Griffith University, Southport, QLD 4215, Australia

^5^ School of Environment and Science, Griffith University, Southport, QLD 4215, Australia

^6^ Department Animal Ecology I and BayCEER, University of Bayreuth, Universitätsstraße 30, 95447 Bayreuth, Germany

Table SI-1: Setups of experiment 1 (December 2018) and 2 (March 2019) with the combination of substrata, MP size, MP concentration, and flow velocity for each channel. The temperature in the circular flow tanks was 10.65 °C in the first and 12.3 °C in the second experiment at the start. This was similar to the temperatures of the River Rhine (Table 2). The temperature dropped on average by 1.8 °C in the first and 1.4 °C in the second experiment after 24 hours.

|  | Channel 1 | Channel 2 | Channel 3 | Channel 4 |
| --- | --- | --- | --- | --- |
| EXPERIMENT 1 (December 2018) | | | | |
| Substrata | BF on rough clay (n = 10)  rough acrylic (n = 3)  smooth acrylic (n = 3) | Smooth clay (n = 4)  Rough clay (n = 4) | BF on rough clay (n = 10)  Smooth clay (n = 3)  Rough clay (n = 3) | Smooth clay (n = 4)  Rough clay (n = 4) |
| Microplastics | 6 µm | 6 µm | 6 µm | 6 µm |
| Concentration | 500 p ml^-1^ | 500 p ml^-1^ | 500 p ml^-1^ | 500 p ml^-1^ |
| Flow velocity | 0.1 m s^-1^ | 0.1 m s^-1^ | 0.2 m s^-1^ | 0.2 m s^-1^ |
| EXPERIMENT 2 (March 2019) | | | | |
| Substrata | BF on rough clay (n = 10) | Rough clay (n = 10) | BF on rough clay (n = 10) | Rough clay (n = 10) |
| Microplastics | 1 µm, 6 µm, 10 µm | 1 µm, 6 µm, 10 µm | 1 µm, 6 µm, 10 µm | 1 µm, 6 µm, 10 µm |
| Concentration | 500 p ml^-1^ | 500 p ml^-1^ | 500 p ml^-1^ | 500 p ml^-1^ |
| Flow velocity | 0.1 m s^-1^ | 0.1 m s^-1^ | 0.2 m s^-1^ | 0.2 m s^-1^ |

Table SI-2: Additional data from the field studies: accumulated absolute and relative number of MPs across all samples in biofilms (A) and controls (B) after 6 months, 12 months, and 18 months regarding size classes.

| **A) BIOFILM** | **6 months** | | **12 months** | | **18 months** | |  | |
| --- | --- | --- | --- | --- | --- | --- | --- | --- |
| **Size class** | **Number of MPs** | **%** | **Number of MPs** | **%** | **Number of MPs** | **%** | **Mean of percentage**  **%** | **Accumulated mean**  **%** |
| **<11 µm** | 220 | 18.33 | 100 | 17.01 | 88 | 23.66 | 19.67 |  |
| **11-50 µm** | 706 | 58.83 | 312 | 53.06 | 176 | 47.31 | 53.07 | 72.73 |
| **51-100 µm** | 144 | 12.00 | 104 | 17.69 | 68 | 18.28 | 15.99 | 88.72 |
| **101-150 µm** | 20 | 1.67 | 44 | 7,48 | 24 | 6.45 | 5.20 | 93.92 |
| **151-300 µm** | 6 | 0.50 | 16 | 2.72 | 16 | 4.30 | 2.51 | 96.43 |
| **301-500 µm** | 4 | 0.33 | 12 | 2.04 | 0 | 0.00 | 0.79 | 97.22 |
| **>500 µm** | 100 | 8.33 | 0 | 0.00 | 0 | 0.00 | 2.78 | 100.00 |
| **sum** | **1200** | **100** | **588** | **100** | **372** | **100** | 100.00 |  |
|  |  |  |  |  |  |  |  |  |
| **B) CONTROL** | **6 months** | | **12 months** | | **18 months** | |  | |
| **Size class** | **Number of MPs** | **%** | **Number of MPs** | **%** | **Number of MPs** | **%** | **Mean of percentage**  **%** | **Accumulated mean**  **%** |
| **<11 µm** | 16 | 11.11 | na | na | 52 | 36.11 | 23.61 |  |
| **11-50 µm** | 96 | 66.67 | na | na | 56 | 38.89 | 52.78 | 76.39 |
| **51-100 µm** | 24 | 16.67 | na | na | 28 | 19.44 | 18.06 | 94.45 |
| **101-150 µm** | 4 | 2.78 | na | na | 0 | 0.00 | 1.39 | 95.84 |
| **151-300 µm** | 4 | 2.78 | na | na | 8 | 5.56 | 4.17 | 100.01 |
| **301-500 µm** | 0 | 0.00 | na | na | 0 | 0.00 | 0.00 | 100.01 |
| **>500 µm** | 0 | 0.00 | na | na | 0 | 0.00 | 0.00 |  |
| **sum** | **144** | **100** | **0** | **0** | **144** | **100** | 100.00 |  |

| A) | 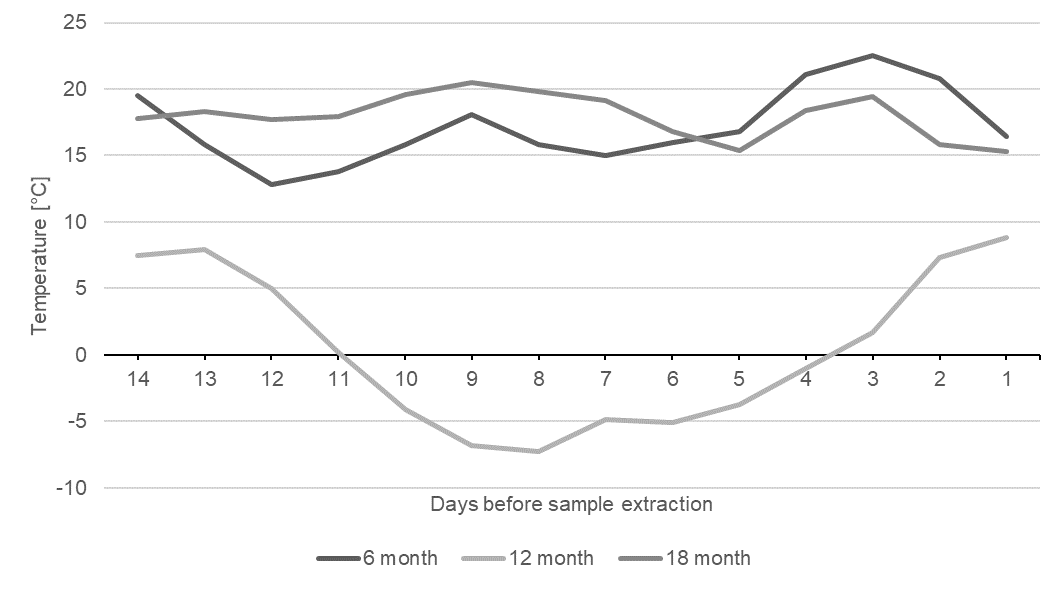 |
| --- | --- |
| B) | 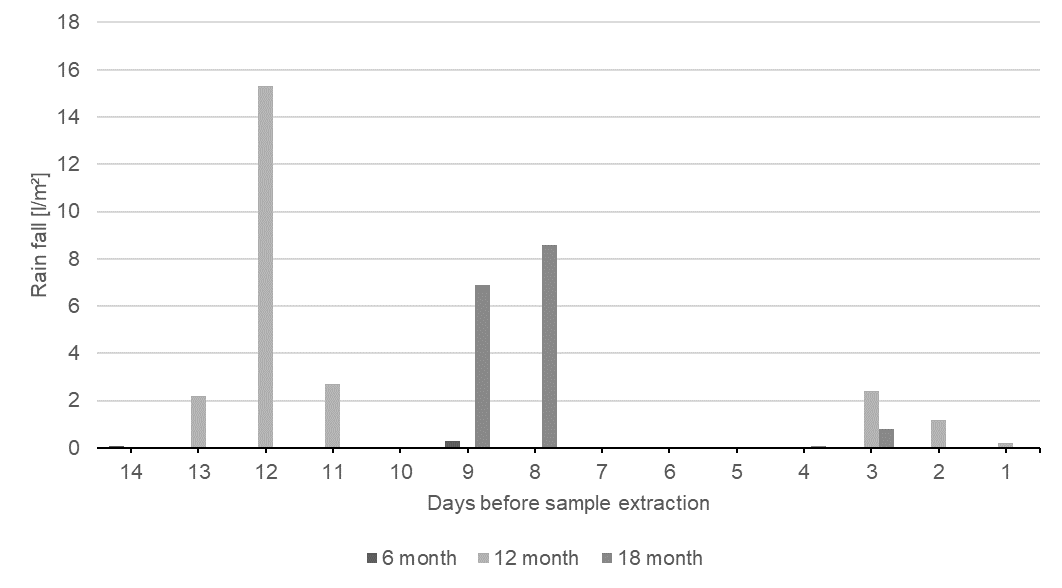 |
| C) | 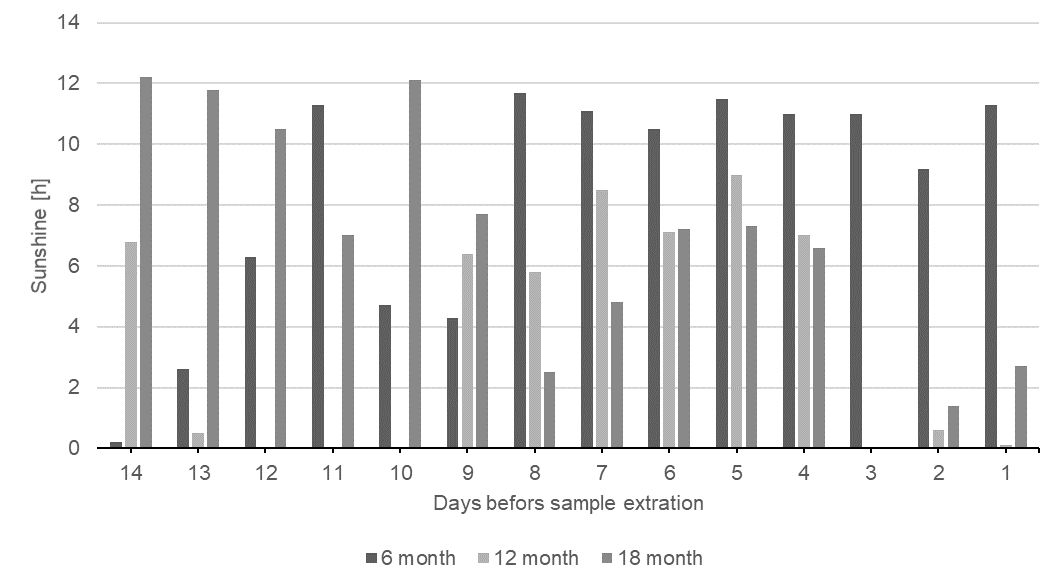 |

Figure SI-1: Weather data for the 14 days prior to sample extraction (6 months: 04.09.2020-17.09.2020; 12 months: 04.02.2021-17.02.2021; 18 months: 04.09.2021-17.09.2021) from the Rhine River in the area of Cologne: A) Temperature, B) Rain fall, and C) Duration of sunshine. Source: www.wetterkontor.de
